# A new species of *Websterinereis* from the Gulf of California and redescription of *Websterinereis foli* (Fauvel, 1930) (Annelida, Nereididae)

**DOI:** 10.3897/zookeys.614.8843

**Published:** 2016-09-01

**Authors:** Jesús Angel de León-González, Eduardo F. Balart

**Affiliations:** 1 Universidad Autónoma de Nuevo León, Facultad de Ciencias Biológicas, Laboratorio de Biosistemátca, Ap. Postal 5”F”, San Nicolás de los Garza, Nuevo León, 66451 Mexico; 2 Laboratorio de Necton y Ecología de Arrecifes, Centro de Investigaciones Biológicas del Noroeste S.C., La Paz, Baja California Sur, 23096 Mexico

**Keywords:** Mexico, Nereididae, new species, polychaetes, taxonomy

## Abstract

A new species of *Websterinereis* Pettibone, 1971, *Websterinereis pettiboneae* **sp. n.** is described from La Paz Bay, Gulf of California, Mexico. This species is similar to *Websterinereis foli* (Fauvel, 1930) in the neuropodial falcigers shape, but can be separated by the tentacular cirri length, notopodial prechaetal lobe shape, and the size of the notopodial dorsal and ventral ligules on posterior parapodia. *Websterinereis foli* is redescribed based upon type material. Additional observations on the inter-annual density variation of *Websterinereis pettiboneae* **sp. n.** during a four-year study are also provided. A key to all species of *Websterinereis* is included.

## Introduction

The family Nereididae de Blainville, 1818 comprises 44 genera which can be separated by a number of characters associated with the eversible pharynx and parapodial structures. *Eunereis* Malmgren, 1965 and *Websterinereis* Pettibone, 1971 are very similar to each other because the pharynx in both has papillae on the oral ring, and there are no paragnaths or papillae on the maxillary ring. These genera can be separated because *Eunereis* has notopodial homogomph falcigers whereas *Websterinereis* presents only notopodial homogomph spinigers. *Websterinereis* was established by Pettibone (1971) and includes four previously described species: *Websterinereis foli* (Fauvel, 1930) from New Caledonia and the Marshall Islands, Pacific Ocean (Reish 1968), *Websterinereis glauca* (Claparède, 1870) from England and along the French Atlantic coast, *Websterinereis tridentata* (Webster, 1879) from the east coast of the United States, and *Websterinereis punctata* (Wesenberg-Lund, 1949) from the Persian Gulf. Most of the records of *Websterinereis* species are restricted to the type locality; the only species with a wider record is *Websterinereis glauca*. These species can be separated into two groups by the presence of anchylosed chaetae, apparently formed by fusion of the blade to the handle. In *Websterinereis punctata* anchylosed chaetae are bifid and restricted to the supra-acicular fascicle of median and posterior neuropodia; in the epitokes of *Websterinereis glauca* there are one or two dark unidentate anchylosed chaetae in a supra-acicular position on the posterior neuropodia. It is noteworthy that in *Websterinereis tridentata* (type species for the genus) and *Websterinereis foli* there are no anchylosed chaetae; *Websterinereis foli* has falcigers with short blades in comparison with those present in *Websterinereis tridentata*, however, a few falcigers of these two species give the appearance of being simple because of blade loss. Of all the described species only *Websterinereis glauca* has reduced notopodial prechaetal lobes present as low ridges, while the other species have larger, more developed lobes, at least in the anterior parapodia.

In this contribution a new species of *Websterinereis* is described which had previously been confused with *Websterinereis foli* due to the morphological similarity of their compound falcigers. In order to further clarify the taxonomic status of this taxon, a re-description of *Websterinereis foli* is provided based on type material. An additional comment on inter-annual density variation, observed during a four-year study, is given for the new species of *Websterinereis*.

## Methods

Specimens of the new species were collected during a long-term study of coral reef recuperation following a tanker vessel grounding in San Lorenzo Channel, La Paz Bay, in the south-western Gulf of California, Mexico. In 2001 thirty concrete and rock, artificial- reef structures were deployed on the bottom of the grounding site. Later, live fragments of the coral *Pocillopora* spp. were attached over the artificial reef surfaces. From 2004 to 2009 a seasonal monitoring survey was carried out aimed at evaluating the structural and ecological recovery of the restored site (Balart et al. 2010). It included samplings of rocky benthic infauna in ten of the artificial reef structures as a proxy for benthic reef community recovery. An area of 0.20 × 0.20 m on a lateral wall in each structure was sampled (0.04 m^2^; total sampled area by survey 0.4 m^2^). This area was fragmented with chisel and hammer and the fragments transferred to polyethylene bags *in situ*. Sorting and taxonomic analysis of formalin-fixed worm material was performed in the Laboratory of Biosystematics (UANL). All identified specimens were deposited in the Polychaete Collection of the Universidad Autónoma de Nuevo León
(UANL). Paratypes were deposited in the Los Angeles County Museum, USA. Terminology of parapodial structures used in this work was taken from the proposed by Bakken and Wilson (2005), elaborated on the basis of previous proposals used by several authors including Hylleberg et al (1986), Hutchings and Reid (1990) and Wilson et al. (2003).

The mean density (individual per m^2^) of the ten artificial reef structures sampled per survey was used to evaluate the variation in abundance of the new species of *Websterinereis* throughout the study period. Relationships between density data and environmental variables were also analyzed.

## Systematic account

### Family Nereididae de Blainville, 1818

#### Genus Websterinereis

Taxon classificationAnimaliaPhyllodocidaNereididae

Pettibone, 1971

Websterinereis Pettibone, 1971: 19.

##### Type species.

*Nereis tridentata* Webster, 1879, by original designation.

##### Diagnosis.

Prostomium sub-pyriform to pentagonal, one pair of frontal antennae, a pair of globose biarticulate palps, and two pairs of eyes of different shape. Peristomium with four pairs of short tentacular cirri. Pharynx with pair of jaws. Maxillary ring unarmed, oral ring with papillae on areas VI and VII-VIII. First two parapodia uniramous, remainder biramous. Notopodium represented by dorsal cirri with dorsal and median ligulae, notopodial prechaetal lobe present on anterior parapodia. Neuropodium with superior and inferior lobe forming prechaetal area; postchaetal lobe subulate to triangular, ventral ligule generally subulate. Ventral cirri short. Notochaetae homogomph spinigers; neurochaetae homogomph and heterogomph spinigers and heterogomph falcigers, those in posterior parapodia with short to long blades; anchylosed chaetae may be present. Pygidium with pair of anal cirri.

#### Websterinereis pettiboneae sp. n.

Taxon classificationAnimaliaPhyllodocidaNereididae

http://zoobank.org/B1311381-9CA7-4F61-8217-8D15EB4823A4

Figs 1 , 2

##### Type material.

Holotype (UANL 7845) and 3 Paratypes (LACM-AHF Poly 9104), San Lorenzo Channel, La Paz Bay, Gulf of California, Mexico, Stn 12 (24°23'11.4"N, 110°18'55.5"W), July 2006.

##### Additional material.

Lorenzo Channel, La Paz Bay, Gulf of California, Mexico, (2 specimens), Stn 28 (24°23'12"N, 110°18'55.1"W), April 2006; (1 specimen), Stn 5 (24°23'12.2"N, 110°18'55.1"W), July 2006; (2 specimens), Stn 27 (24°23'12"N, 110°18'54.9"W), July 2006; (1 specimen), Stn 1 (24°23'12.8"N, 110°18'54.2W), October 2006; (4 specimens, 2 epitokes), Stn 3 (24°23'12.8"N, 110°18'54.8"W), October 2006; (1 specimen), Stn 5 (24°23'12.2"N, 110°18'55.1"W), October 2006; (12 specimens), Stn 9 (24°23'11.7N, 110°18'55.4"W), October 2006; (1 epitokous specimen), Stn 12 (24°23'11.4"N, 110°18'55.5"W), October 2006; (1 specimen), Stn 13 (24°23'11.5"N, 110°18'54.8"W), October 2006; (3 specimens), Stn 3 (24°23'12.8"N, 110°18'54.8"W), 3 October 2007; (3 specimens), Stn 9 (24°23'11.7N, 110°18'55.4"W), 3 October 2007; (1 specimen), Stn 13 (24°23'11.5"N, 110°18'54.8"W W), 3 October 2007.

##### Description.

**Figure 1.**
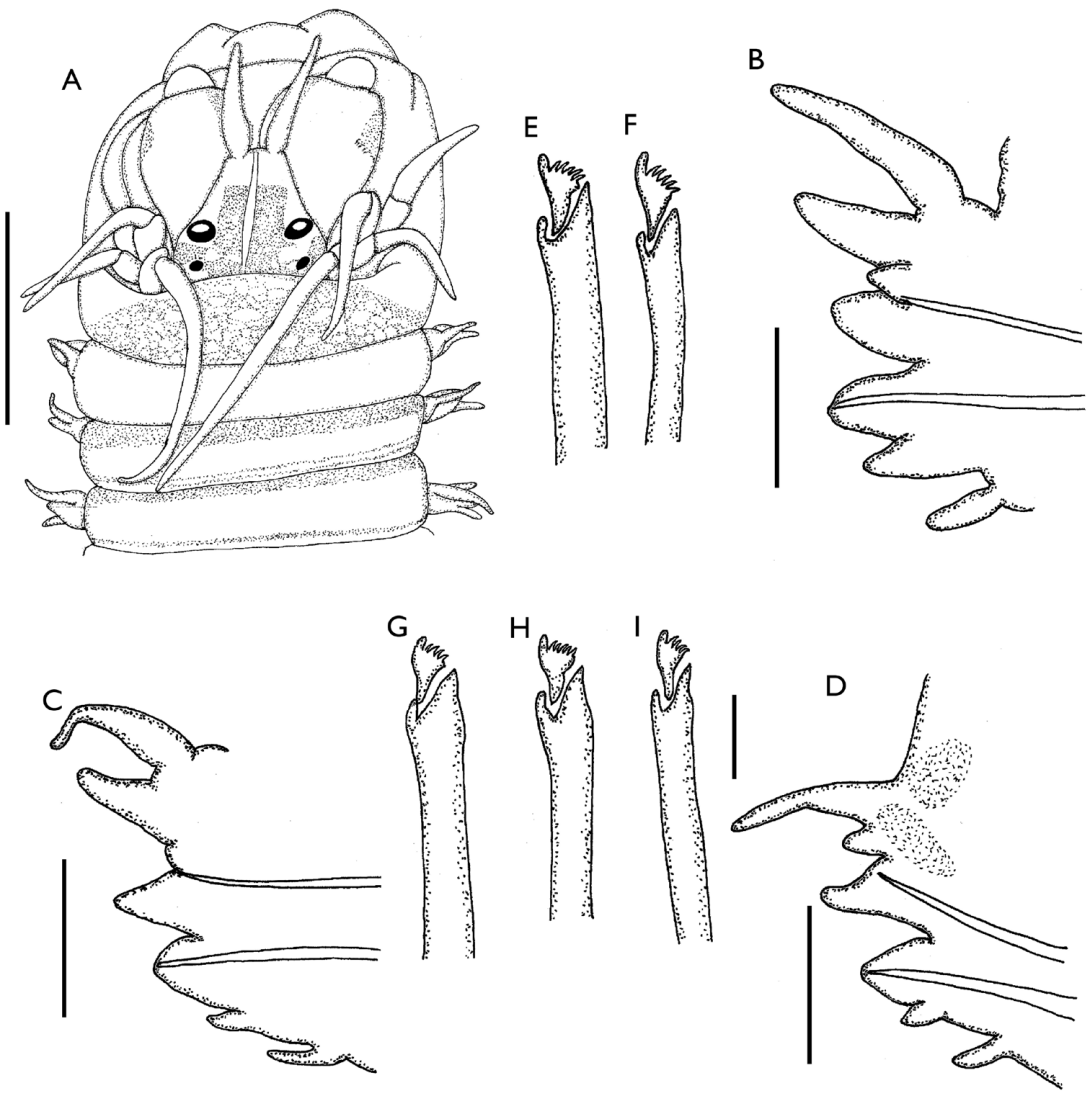
*Websterinereis pettiboneae* sp. n. **A** Anterior end, dorsal view **B–D** Parapodia of chaetigers 10, 26 and 42, anterior view **E** Supra-acicular neuropodial heterogomph falciger, chaetiger 10 **F** Infra-acicular neuropodial heterogomph falciger, chaetiger 10 **G** Supra-acicular neuropodial heterogomph falciger, chaetiger 26 **H** Infra-acicular neuropodial heterogomph falciger, chaetiger 26 **I** Supra-acicular neuropodial heterogomph falciger, chaetiger 42. Scale bars: **A** = 0.5 mm; **B–D** = 100 µm; **E–I** = 10 µm.

Holotype complete with restricted blackish pigmentation (Fig. 1A); prostomium with anteriorly truncate, extended dark area, leaving pale mid-dorsal thin band, not reaching anterior prostomial margin; palpophores with some pigmentation over external, subdistal surfaces; tentacular segment with continuous dorsal transverse wide band, reduced to progressively thinner bands along anterior and posterior segmental margins.

Body 12 mm long, 0.8 mm wide including parapodia, with 60 chaetigers. Prostomium subpyriform, longer than wide. Two pairs of black oval eyes in rectangular arrangement, distal pair with larger lens than proximal pair. Antennae tapered, extended beyond tips of palpostyles. Palps and palpostyles globose. One apodous anterior segment, 1.5 times longer than first chaetiger. Tentacular cirri short, tapered, longest reaching chaetiger 3 (Fig. 1A).

Pharynx with brown jaws, each with six teeth. Maxillary ring lacking paragnaths or papillae; oral ring with subconical papillae in area VI, and five globose papillae in line along areas VII–VIII.

Parapodia of first two chaetigers uniramous, remainder biramous. In anterior parapodia notopodia with subulate dorsal ligules, notopodial prechaetal lobes short triangular, and ventral ligules subtriangular, rounded distally; neuropodia with postchaetal lobes distally rounded, superior and inferior lobes not distinct, ventral ligules subulate. Dorsal cirri inserted basally, four times longer than ventral cirri, and 1.4 times than notopodial dorsal ligule (Fig. 1B). Median parapodia with digitiform dorsal ligules, notopodial prechaetal lobes reduced to small ridge; ventral ligules triangular; neuropodial postchaetal lobes subconical, ventral ligules reduced to small subulate protuberance. Dorsal cirri three times longer than ventral cirri (Fig. 1C). Posterior parapodia with dorsal ligules reduced, half as long as those of median parapodia, notopodial prechaetal lobes absent, ventral ligules triangular; neuropodial postchaetal lobes subtriangular, ventral ligules reduced, conical. Dorsal cirri 2.5 times longer than ventral cirri, with red pigmented glandular areas (Fig. 1D).

Anterior parapodia with notochaetae represented by four supra-acicular homogomph spinigers; supra-acicular neurochaetae include two homogomph spinigers and two heterogomph falcigers with thick handle and short triangular blade (Fig. 1E); infra-acicular neurochaetae represented by five heterogomph falcigers (Fig. 1F). Median parapodia with supra-acicular notochaetae represented by two supra-acicular homogomph spinigers; supra-acicular neurochaetae include two homogomph spinigers and one heterogomph falciger with thick handle and short triangular blade (Fig. 1G), infra-acicular neurochaetae represented by four heterogomph falcigers (Fig. 1H). Posterior parapodia with notochaetae represented by two supra-acicular heterogomph spinigers; supra-acicular neurochaetae with a single heterogomph falciger (Fig. 1I); infra-acicular neurochaetae three heterogomph falcigers, similar to supra-acicular falcigers.

Pygidium with terminal anus and two anal cirri.

**Figure 2.**
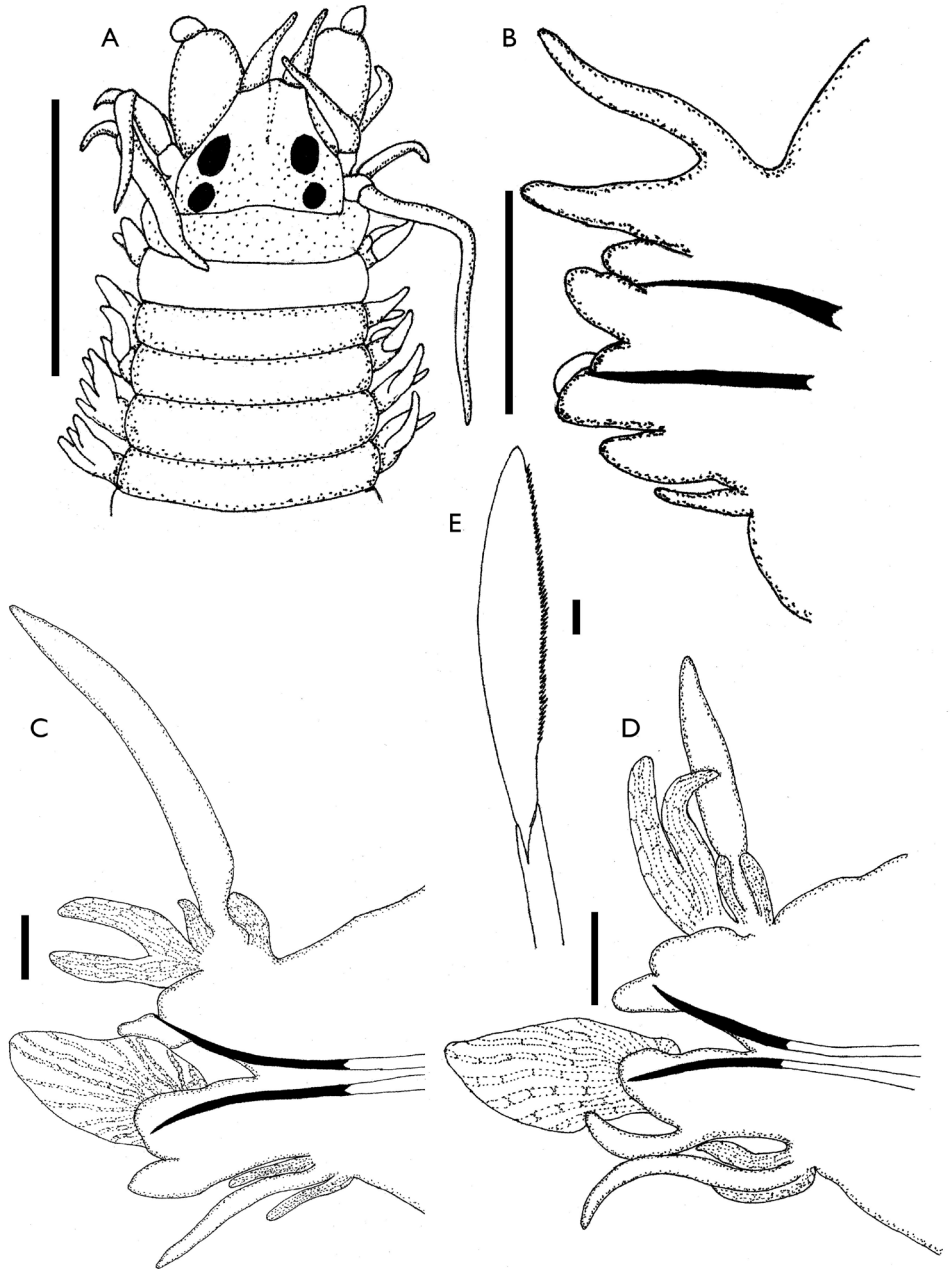
*Websterinereis pettiboneae* sp. n. (epitoke). **A** Anterior end, dorsal view **B–D** Parapodia of chaetigers 10, 30 and 43, anterior view **E** Natatory chaeta. Scale bars: **A** = 0.5 mm; **B–D** = 100 µm; **E** = 10 µm.

**Epitokous female.** Best preserved specimen with 64 chaetigers, 9 mm long and 0.5 mm wide (excluding parapodia). Prostomium pentagonal, wider than longer, with frontal median dorsal groove. Antennae minute, shorter than anterior end of palpi. Two pairs of eyes in trapezoidal arrangement, anterior pair enlarged, oval in shape, posterior pair rounded in shape. Biarticulate palps globose, each with spherical palpostyle. Tentacular ring with four pairs of tentacular cirri, posterodorsal pair extending back to anterior margin of sixth chaetiger (Fig. 2 A). Pharynx equal to non-epitokous specimens.

Body divided into unmodified anterior region and a heteronereidid region; parapodia of first 19 chaetigers similar of those of atokous specimens (Fig. 2B). Parapodia of heteronereidid region moderately compressed. Notopodia formed by dorsal cirrus accompanied by two basal lobes, a small notopodial dorsal ligule, and subulate ventral ligule; with two enlarged postchaetal lobes. Neuropodia with superior and inferior lobes fused, foliose postchaetal lamellae present; neuropodial ventral ligule subtriangular in median parapodia and digitate in posterior parapodia. Ventral cirri long and thin, accompanied by two basal lobes (Fig. 2C–D). Last eleven chaetigers unmodified. Normal chaetae replaced on chaetigers 20 by natatory chaetae with broad, paddle-shaped appendages, inner margin slightly denticulate (Fig. 2E). Anterior parapodia with four homogomph spinigers in notopodial supra-acicular position; neuropodia with three supra-acicular homogomph spinigers and one heterogomph falciger; infra-acicular chaetae five heterogomph falcigers. Median parapodia with two homogomph spinigers and 15–18 natatory chaetae in notopodial supra-acicular position; neuropodial infra-acicular chaetae two homogomph spinigers and two heterogomph falcigers, with 14–15 natatory chaetae. Posterior parapodia with two heterogomph falcigers and 13–14 natatory chaetae in notopodial supra-acicular position; neuropodial infra-acicular chaetae one homogomph spiniger, one heterogomph falciger, and 18–20 natatory chaetae.

Pygidium similar to those of atokous specimens.

##### Etymology.

Specific name is in honor of Marian H. Pettibone for her great work on increasing the knowledge of polychaetes.

##### Remarks.

*Websterinereis pettiboneae* sp. n. resembles *Websterinereis foli* in the shape of the compound falcigers, although there is greater variation in the shape of compound falcigers in *Websterinereis foli*. These species differ in the following features: *Websterinereis pettiboneae* has longer tentacular cirri reaching chaetiger 3, notopodial prechaetal lobes are triangular, and notopodial dorsal and ventral ligule are progressively smaller in posterior parapodia. In *Websterinereis foli* the longest tentacular cirri reaches chaetiger one, with a thin, cirriform prechaetal notopodial lobe inserted at the base of the notopodial ventral ligule, and the dorsal and ventral ligule increasing slightly in posterior parapodia.

##### Distribution.

*Websterinereis pettiboneae* sp. n. is known only from Canal de San Lorenzo, La Paz Bay, Gulf of California, Mexico.

##### Density.

**Figure 3.**
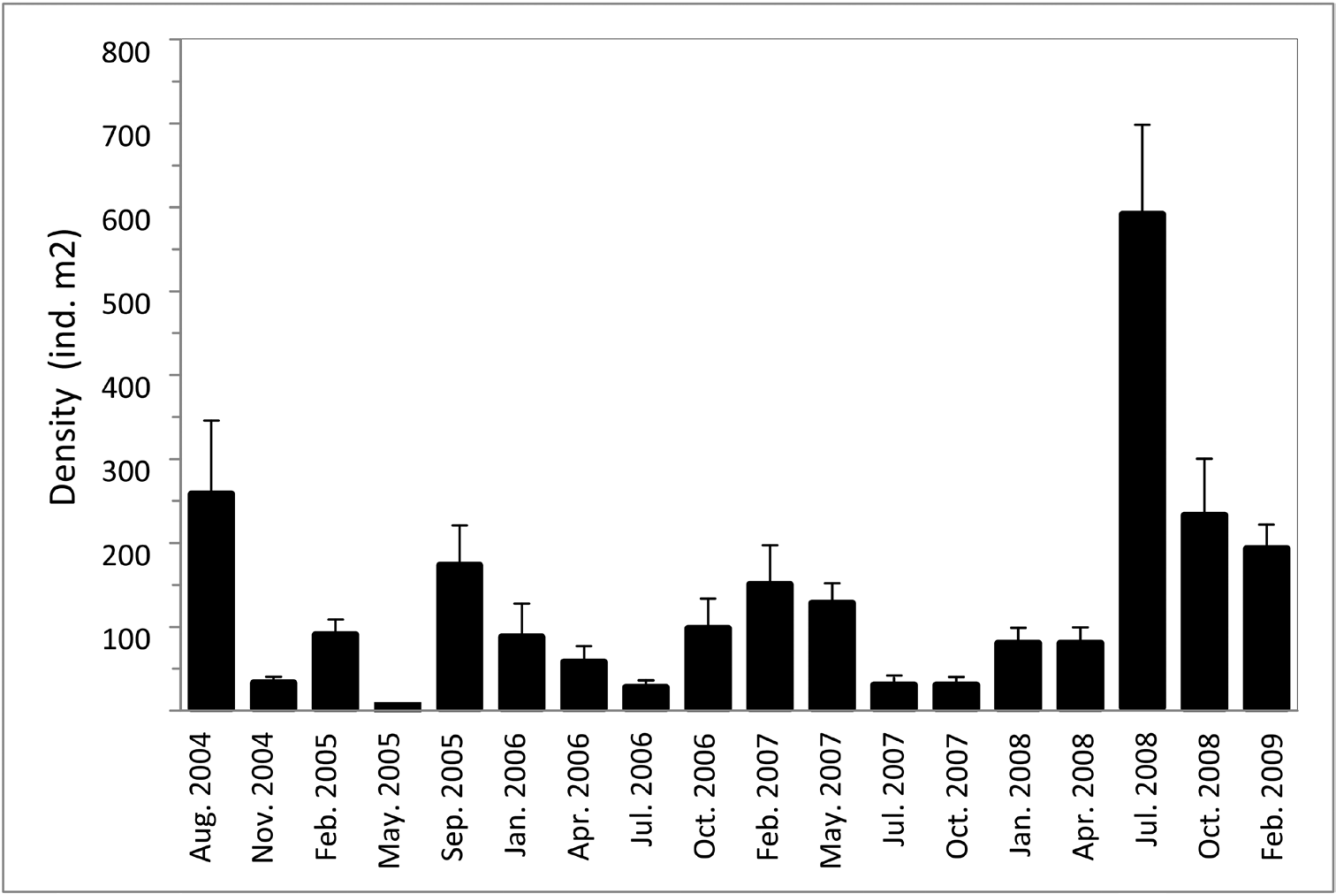
Mean density (individual per m^2^ ± ES) of *Websterinereis pettiboneae* sp. n. in the restored area of the reef at San Lorenzo Channel, Bahía de La Paz, southern Gulf of California.

The mean density of *Websterinereis pettiboneae* sp. n. varied from 2.5 ±2.5 individuals per m^2^, in May 2005, to a maximum of 587.5 ±110.8 individuals per m^2^ recorded in July 2008 (Fig. 3). In general, densities were higher during the warmer and colder months and the lower densities between them; it suggests two peaks of recruitment for this species in the rocky reefs of southern Gulf of California. However, no significant relationship between temperature or salinity with worm density could be established.

#### Websterinereis foli

Taxon classificationAnimaliaPhyllodocidaNereididae

(Fauvel, 1930)

Fig. 4

Leptonereis foli Fauvel, 1930: 529, fig. 3.
Websterinereis foli : Pettibone 1971: 23, figs 10–11; Hutchings and Reid 1990: 91; Pamungkas and Glasby 2015: 19.
Nicon sp. Martens et al. 1995: 17, figs 20–24.

##### Type material.

Holotype of *Leptonereis foli* (MNHN-685), Île des Pins, New Caledonia, 1 Jan. 1928, leg. Mme A. Pruvot-Fol.

##### Redescription.

Holotype incomplete, in two fragments; anterior fragment 15 mm long, 1.1 mm wide including parapodia at chaetiger 10, with 54 chaetigers; medial region fragment 2.5 mm long, 0.7 mm wide including parapodia, with 8 chaetigers.

Pigmentation blackish; prostomium with longitudinal narrow band throughout its length, leaving thin pale mid-dorsal line, pale subtriangular area around the anterior eyes, and pale semicircular area around posterior eyes; palpophores pale; tentacular segment with solid blackish dorsal pigmentation, laterally pale, reduced to wide dorsal longitudinal band on first chaetiger.

**Figure 4.**
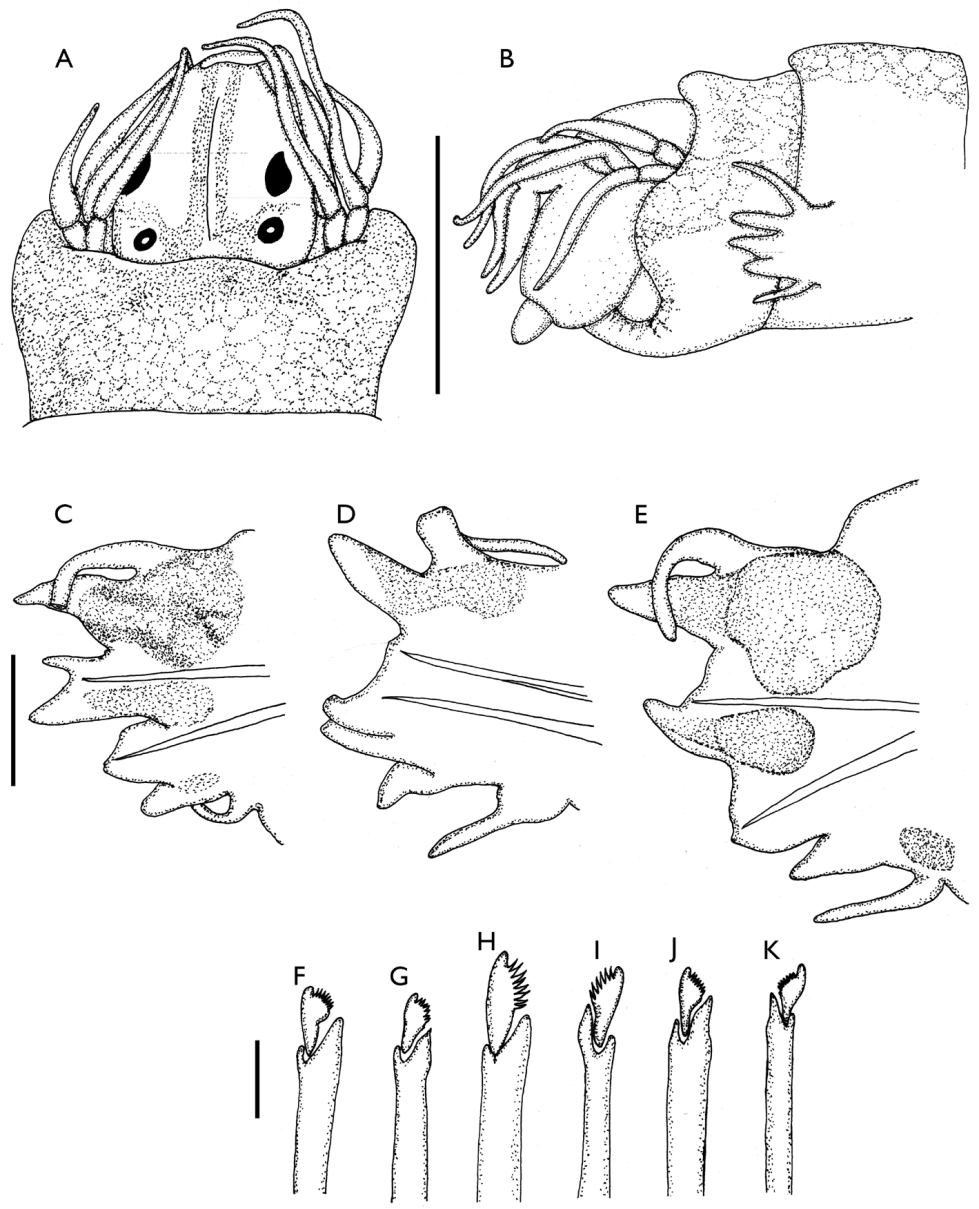
*Websterinereis foli*. Holotype (MNHN-685). **A** Anterior end, dorsal view **B** Anterior end, lateral view **C–E** Parapodia of chaetigers 10 (dorsal cirri incomplete), 20 (dorsal cirrus folded backwards) and 38, anterior view **F** Supra-acicular neuropodial heterogomph falciger, chaetiger 10 **G** Infra-acicular neuropodial heterogomph falciger, chaetiger 10 **H** Supra-acicular neuropodial heterogomph falciger, chaetiger 20 **I** Infra-acicular neuropodial heterogomph falciger, chaetiger 20 **J** Supra-acicular neuropodial heterogomph falciger, chaetiger 38 **K** Infra-acicular neuropodial heterogomph falciger, chaetiger 38. Scale bars: **A–B** = 0.5 mm; **C–E** = 100 µm; **F–K** = 10 µm.

Prostomium subpentagonal, longer than wide, with slight depression along anterior half. Two pairs of black eyes, distal ones reniform larger than proximal ones which are rounded and show lenses. Antennae tapered, not reaching tips of palpostyles; palps globose, palpostyles conical; both antennae and palps directed ventrally as result of an artifact of fxation. One apodous anterior segment, shorter than first chaetiger. Tentacular cirri short, tapered, longest pair reaching chaetiger one (Fig. 4 A–B).

Pharynx with single jaw (left one lost), thin, with seven well-developed teeth. Maxillary ring without paragnaths or papillae; oral ring with triangular papillae on area VI, area V lacking papillae, areas VII–VIII, with seven rounded papillae.

Parapodia of first two chaetigers, uniramous, thereafter biramous. Anterior notopodia with triangular dorsal and ventral ligules, with thin notopodial prechaetal lobe inserted at base of notopodial ventral ligule; neuropodial postchaetal lobes subtriangular, superior and inferior lobes absent, neuropodial ventral ligules subconical. Dorsal cirri inserted basally, longer than ventral cirri. With large glandular area on notopodia, and smaller one on base of neuropodial ventral ligule (Fig. 4C). Median parapodia with subulate dorsal ligules, prechaetal lobes reduced to small rounded protuberances, ventral ligules triangular; neuropodial postchaetal lobes subconical, ventral ligules subtriangular. Dorsal cirri longer than ventrals, both inserted basally. Glandular area present in supra-acicular region (Fig. 4D). Posterior parapodia with triangular dorsal ligules, notopodial prechaetal lobes absent, ventral ligules triangular; neuropodial postchaetal lobes broad, wider than long, ventral ligules subtriangular. Dorsal and ventral cirri subequal (Fig. 4E).

Anterior parapodia with four homogomph spinigers in supra-acicular notochaetae; supra-acicular neurochaetae include three homogomph spinigers and two heterogomph falcigers with thick handle and short triangular blades (Fig. 4F); infra-acicular neurochaetae seven heterogomph falcigers with blades diminishing gradually ventrally (Fig. 4G). Median parapodia with three homogomph spinigers in supra-acicular notochaetae; supra-acicular neurochaetae three homogomph spinigers and two heterogomph falcigers with thick handle and oval blades (Fig. 4H); infra-acicular neurochaetae seven heterogomph falcigers with thin handles and triangular blades (Fig. 4I). Posterior parapodia with five homogomph spinigers in supra-acicular notochaetae; supra-acicular neurochaetae include two homogomph spinigers and one heterogomph falciger (Fig. 4J); infra-acicular neurochaetae six heterogomph falcigers (Fig. 4K). Pygidium unknown.

##### Remarks.

After reviewing the holotype of *Websterinereis foli* some differences were noted from the description by Pettibone (1971). In her description of *Websterinereis foli* Pettibone combined her observations of the holotype of *Leptonereis foli* Fauvel, 1930 and the holotype and paratypes of *Ceratocephala corallicola* Reish, 1968. This explains why her description begins with the measurements of a specimen that does not match the holotypes of either species, as 20 mm long and up to 112 chaetigers. Since Pettibone (1971) mixed morphological features of these two species originally described from very distant localities we have restricted our redescription of *Websterinereis foli* to only the specimen that Fauvel (1930) designated as the holotype.

##### Distribution.

Central Pacific (New Caledonia, Marshall Islands), Australia (Western Australia, New South Wales, South Australia, Lizard Islands), Indonesia.

### Key to *Websterinereis* species modified from Pettibone (1971)

**Table:** 

1	Anterior parapodia with notopodial prechaetal lobes small, inconspicous; unidentate ankylosed chaetae present in epitokous stage	***Websterinereis glauca***
–	Anterior parapodia with notopodial prechaetal lobes distinct	**2**
2	Neuropodial falcigers with long blades	**3**
–	Neuropodial falcigers with short blades	**4**
3	Longest tentacular cirri reach chaetiger 6; notopodial prechaetal lobe present along body; bidentate ankylosed chaetae present on median and posterior parapodia	***Websterinereis punctata***
–	Longest tentacular cirri reach chaetiger 2 (1-4); notopodial prechaetal lobe present only on anterior region; ankylosed chaetae absent	***Websterinereis tridentata***
4	Longest tentacular cirri reach chaetiger 1; notopodial prechaetal lobe thin, digitiform; notopodial dorsal and ventral ligules increasing in length slightly on posterior parapodia	***Websterinereis foli***
–	Longest tentacular cirri reach chaetiger 3; notopodial prechaetal lobe triangular; notopodial dorsal and ventral ligule are progressively smaller in posterior parapodia	***Websterinereis pettiboneae***

## Supplementary Material

XML Treatment for Websterinereis

XML Treatment for Websterinereis pettiboneae

XML Treatment for Websterinereis foli
